# Genetic Variation Associated with Differential Educational Attainment in Adults Has Anticipated Associations with School Performance in Children

**DOI:** 10.1371/journal.pone.0100248

**Published:** 2014-07-17

**Authors:** Mary E. Ward, George McMahon, Beate St Pourcain, David M. Evans, Cornelius A. Rietveld, Daniel J. Benjamin, Philipp D. Koellinger, David Cesarini, The Social Science Genetic Association Consortium, George Davey Smith, Nicholas J. Timpson

**Affiliations:** 1 MRC Integrative Epidemiology Unit at the University of Bristol, School of Social and Community Medicine, University of Bristol, Bristol, United Kingdom; 2 School of Oral and Dental Sciences, University of Bristol, Bristol, United Kingdom; 3 School of Experimental Psychology, University of Bristol, Bristol, United Kingdom; 4 University of Queensland Diamantina Institute, Translational Research Institute, Brisbane, Queensland, Australia; 5 Department of Applied Economics, Erasmus School of Economics, Erasmus University Rotterdam, Rotterdam, Netherlands; 6 Department of Epidemiology, Erasmus Medical Center, Rotterdam, Netherlands; 7 Department of Economics, Cornell University, Ithaca, New York, United States of America; 8 Amsterdam Business School, University of Amsterdam, Amsterdam, Netherlands; 9 Center for Experimental Social Science, Department of Economics, New York University, New York, New York, United States of America; 10 Division of Social Science, New York University Abu Dhabi, Abu Dhabi, United Arab Emirates; 11 Research Institute of Industrial Economics, Stockholm, Sweden; University of North Carolina, United States of America

## Abstract

Genome-wide association study results have yielded evidence for the association of common genetic variants with crude measures of completed educational attainment in adults. Whilst informative, these results do not inform as to the mechanism of these effects or their presence at earlier ages and where educational performance is more routinely and more precisely assessed. Single nucleotide polymorphisms exhibiting genome-wide significant associations with adult educational attainment were combined to derive an unweighted allele score in 5,979 and 6,145 young participants from the Avon Longitudinal Study of Parents and Children with key stage 3 national curriculum test results (SATS results) available at age 13 to 14 years in English and mathematics respectively. Standardised (z-scored) results for English and mathematics showed an expected relationship with sex, with girls exhibiting an advantage over boys in English (0.433 SD (95%CI 0.395, 0.470), p<10^−10^) with more similar results (though in the opposite direction) in mathematics (0.042 SD (95%CI 0.004, 0.080), p = 0.030). Each additional adult educational attainment increasing allele was associated with 0.041 SD (95%CI 0.020, 0.063), p = 1.79×10^−04^ and 0.028 SD (95%CI 0.007, 0.050), p = 0.01 increases in standardised SATS score for English and mathematics respectively. Educational attainment is a complex multifactorial behavioural trait which has not had heritable contributions to it fully characterised. We were able to apply the results from a large study of adult educational attainment to a study of child exam performance marking events in the process of learning rather than realised adult end product. Our results support evidence for common, small genetic contributions to educational attainment, but also emphasise the likely lifecourse nature of this genetic effect. Results here also, by an alternative route, suggest that existing methods for child examination are able to recognise early life variation likely to be related to ultimate educational attainment.

## Introduction

Although evidence from twin and family studies suggests that psycho-social traits such as educational attainment are moderately heritable [1]–[6], identifying genetic variants reliably associated with such traits has proven particularly challenging. This is likely due to the use of samples that are too small and has been responsible for numerous false positives [2], [7], [8].

In efforts to address this, Rietveld et al. conducted a genome-wide association study (GWAS) of educational attainment in 126,559 individuals, which is much larger than previous social-science genetic studies [9]. This study identified three single nucleotide polymorphisms (SNPs) (rs9320913, rs11584700 and rs4851266) which were associated with educational attainment in adults. These variants are located in independent loci and whilst the mechanism of their association is unknown, they have been advanced as possible targets for further studies of either biological function or where intermediate phenotypes for educational attainment are available.

Effect sizes at these three confirmed signals are small (∼0.02% of variance explained by the strongest of these – equating to ∼1 month in total schooling experience per allele) and sit in a wider context where the genome-wide linear polygenic score from all SNPs accounts for only around 2% of variation in educational attainment. This architecture compares to that seen in other polygenic complex traits such as BMI and height which have single largest common genetic effects explaining up to 0.4% of observed variance and which have total common variant contributions that are far stronger [10]–[12]. Despite this, these results are the first to report a replicable genetic association with educational attainment and merit further dissection.

A natural extension to Rietveld et al. [9] is to follow-up the initial analysis in a collection whose phenotypic measurement is more intermediate or marks more formative features to ultimate educational attainment and in a study where known population characteristics contribute to the validation of the original signal of association. Whilst often not available in large samples, an ideal situation in this case would be to reassess the original genetic association in an ethnically homogeneous, large, population-based collection with a standardised method for the assessment of educational performance and potentially one from an earlier part of the lifecourse. Not only would this provide a form of validation for the original association signal, but improved measurement would theoretically heighten analytical power. Further to this, association would also provide an alternative source of evidence suggesting that early life assessments are able to capture meaningful information about the likely formative events going on to be important for ultimate educational attainment.

This investigation set out to generate an allele score from genome-wide significant SNPs derived from the original association study for educational attainment and assess its association with refined measures of educational performance. To do this, this investigation derived this allele score (also known as a genetic risk score) and assessed whether this score-based summary of genetic variation is associated with educational attainment measured by key stage 3 national curriculum test results at age 13 to 14 years. The data used comes from an ethnically homogenous collection from the South West of the UK, the Avon Longitudinal Study of Parents and Children (ALSPAC).

## Results

The mean English and mathematics non-standardised scores recorded within the ALSPAC collection were level 5.63 (SD = 1.09) for English and level 6.18 (SD = 1.32) for mathematics respectively. These data were available for 10,323 English scores and 10,683 mathematics scores at age 13 to 14 years. The total number of children with data available for the analyses (i.e. with genotypic data, covariables and outcomes) was 5,979 for English, and 6,145 for mathematics.

**Figure 1:**
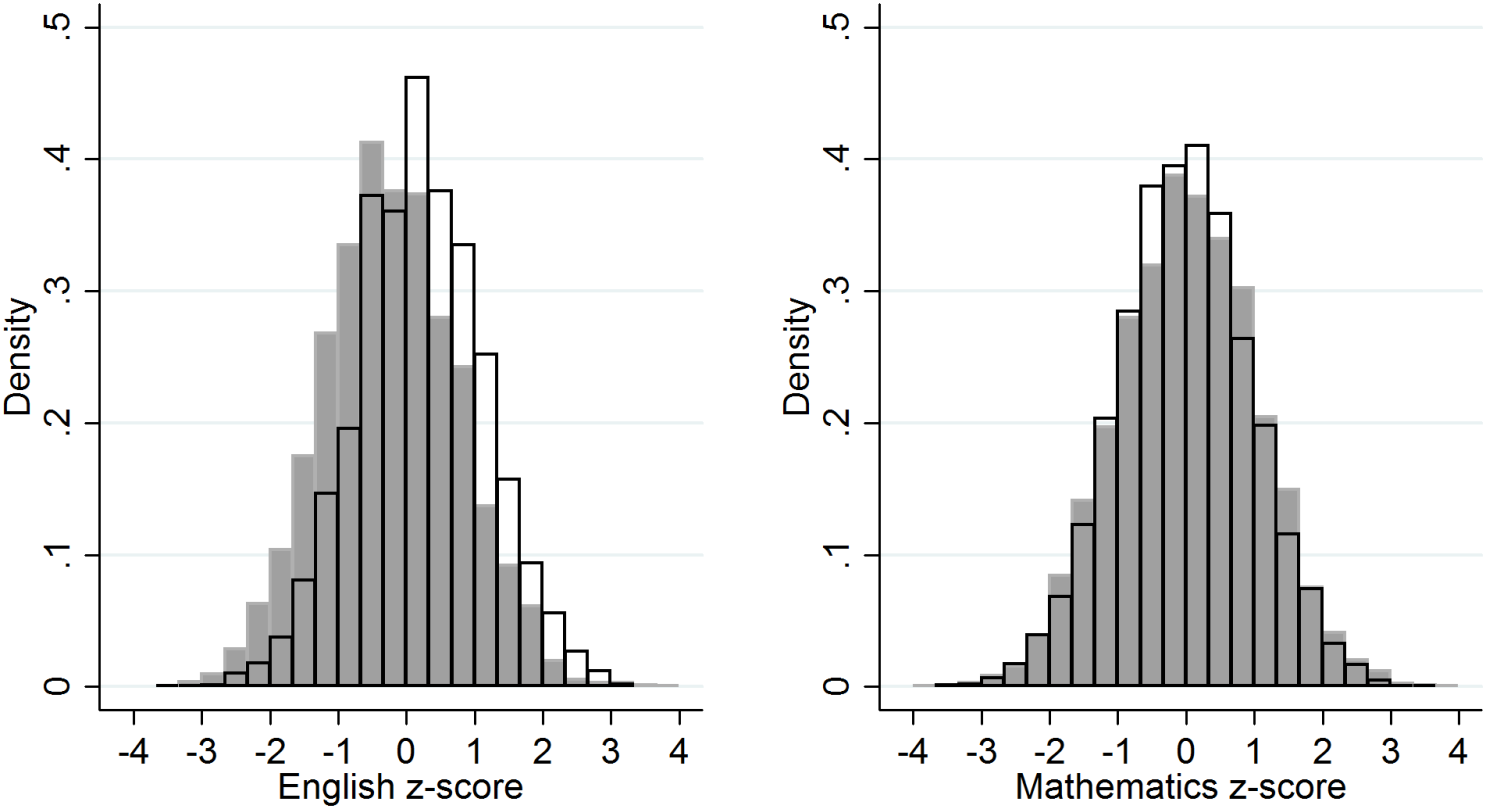
Overlay histograms showing z-scores for English and mathematics stratified by sex within the ALSPAC study. Boys’ results in grey; girls’ results in white. Girls exhibit an average 0.433 SD (95%CI 0.395, 0.470), p<10^−10^ advantage over boys in English, and attain more similar exam results in mathematics with boys exhibiting an average 0.042 SD (95%CI 0.004, 0.080), p = 0.0303 advantage over girls in mathematics.

English and mathematics z-scores showed an expected relationship with sex, with girls exhibiting an advantage over boys in English on average (0.433 SD (95%CI 0.395, 0.470), p<10^−10^) and attaining more similar exam results to boys in mathematics (boys higher than girls, 0.042 SD (95%CI 0.004, 0.080), p = 0.0303) (**Figure 1**).

**Figure 2:**
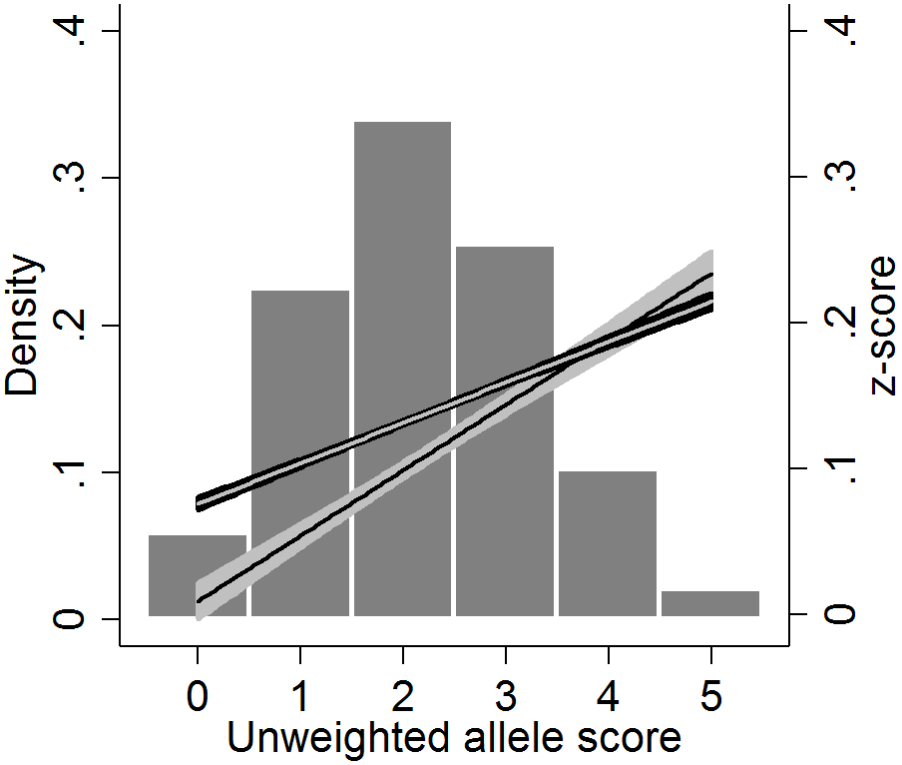
Histogram of allele score, with linear relationships between SATS z-scores and the allele score superimposed. The unweighted allele score is created from three SNPs rs9320913, rs11584700 and rs4851266. Each unit increase in the allele score corresponds to an individual having an additional educational attainment increasing allele. The density for the allele score taking the value 6 is 0.0016, which is too small to be visible in this figure. The linear relationships with 95%CIs from our regressions of SATS z-scores on allele score are superimposed. The English regression is represented by a black line with grey 95%CI, and mathematics by a grey line with black 95%CI.

The SNPs rs9320913, rs11584700 and rs4851266 were observed within the ALSPAC sample set at minor allele frequencies of 0.483, 0.216 and 0.393 respectively. These all passed quality control for imputation and were imputed with RSQR scores of >0.99 [13]. Mean unweighted allele score was 2.19 and attended an approximately normal distribution (**Figure 2**).

English z-score was associated with the unweighted allele score with each additional educational attainment increasing allele being associated with a 0.041 SD increase in SATS z-score (95%CI 0.020, 0.063), p = 0.0002. Whilst there was weaker evidence of association for mathematics, each additional educational attainment increasing allele was associated with a 0.028 SD increase in SATS z-score (95%CI 0.007, 0.050), p = 0.0103 (**Figure 2**).

A proportion (58.3%) of participants in this study had mothers involved in the original discovery study for adult educational attainment [9]. Neither taking into account maternal allele score in analyses nor looking only at offspring whose mothers were not part of the original discovery study had a substantive effect on the results shown above (**Table 1**). The relationships were also maintained when controlling for the first four principal components (PC) of genetic population structure in ALSPAC. We found no evidence for differences in the observed effects by sex. Results from sex-specific analyses can be found in **Table S2**.

**Table 1:** Relationships between SATS z-scores and unweighted allele score within the ALSPAC study.

Regression model	Beta	95%CI	P-value	Number of observations
**English z-score**				
OLS of English z-score on child’s allele score(all children)	0.041	0.020, 0.063	0.0002	5,979
OLS of English z-score on child’s allele score, adjusting for maternal allele score (all children)	0.041	0.011, 0.071	0.0076	4,008
OLS of English z-score on child’s allele score(children without maternal genome-wide data)	0.037	0.0003, 0.074	0.0483	1,971
OLS of English z-score on child’s allele score(all children), controlling forfirst four PCs of genetic population structure in ALSPAC	0.041	0.020, 0.063	0.0002	5,979
**Mathematics z-score**				
OLS of mathematics z-score on child’s allele score(all children)	0.028	0.007, 0.050	0.0103	6,145
OLS of mathematics z-score on child’s allele score,adjusting for maternal allele score(all children)	0.040	0.010, 0.071	0.0094	4,106
OLS of mathematics z-score on child’s allele score(children without maternal genome-wide data)	0.015	−0.022, 0.052	0.4286	2,039
OLS of mathematics z-score on child’s allele score(all children), controlling for first four PCs ofgenetic population structure in ALSPAC	0.028	0.007, 0.050	0.0102	6,145

All models include sex and age as covariables.

SNP-specific effects were underpowered given the relatively small amount of variance explained by singular SNPs. However these analyses showed evidence for an association between rs9320913 and both the English and mathematics z-scores (**Table S3**).

## Discussion

Our results suggest that there is an association between a child’s educational performance at age 13 to 14 years and their unweighted allele score created from the three SNPs (rs9320913, rs11584700 and rs4851266) found to be associated with adult educational attainment by Rietveld et al. [9]. This is of particular interest as we are not only able to reposition the phenotype initially used in the discovery to one of intermediate nature (or process during the lifecourse), but we have been able to demonstrate that in a homogeneous population using commonly available, standardised, exam results. Relationships between genotypic variation and SATS z-scores were consistent across a series of testing scenarios taking into account the presence of mothers in the original data set.

This work sits in a wider context of research attempting to understand the aetiology of extremely complex traits which have traditionally been recognised predominantly as social phenomena. Educational attainment is critically important as it is strongly associated with social and health related outcomes and there is a recognised gradient in relationships between education and health status [9], [14]–[17]. To this complicated and controversial field, estimates of the genetic contribution to educational attainment have suggested that up to 40% of the variance in existing measures may be explained by genetic factors [3], [14], [18]. Some twin studies [4]–[6] have suggested that genetic variation accounts for up to 60% of the variation in educational attainment, though these high heritability estimates may be inflated by genetic interactions [19].

Previous work addressing this directly attempted to collect a harmonized measure of educational attainment in large numbers to try to unpick the contribution of common genetic variation [9]. This work coded study-specific measurements pertinent to education using the International Standard Classification of Education (1997) scale [20] and derived variables marking an individual’s years of schooling or whether college education had been completed. Whilst successful in identifying genetic correlates, this work is of course limited by the distal nature of the measurements employed. Work here has been able to give evidence that there is likely to be a quantifiable genetic contribution to not only attained educational experience, but performance within childhood educational experience.

A 1 SD increase in the English and mathematics z-scores is approximately equivalent to increases of 1.09 levels and 1.32 levels in the non-standardised English and mathematics scores respectively. This corresponds to a child’s non-standardised English score increasing by ∼0.045 levels and their non-standardised mathematics score increasing by ∼0.038 levels for each additional educational attainment increasing allele. To put this into context, if a child’s English level increases by 2 national curriculum levels between the ages of 11 and 14, then an increase of 0.045 levels is approximately equivalent to the increase that would be expected over 3.5 weeks within this period. Put another way, these differences approximate to a tenth of that seen across the sexes for performance in English at this age.

Whilst it is not possible to make direct comparisons between the effect sizes reported here and those elsewhere owing to differing phenotypic measures, it is possible to make some comparison of variance explained. For total years of schooling, the strongest reliable genetic association concerning a single SNP was that of rs9320913 which, in a previous study, explained 0.02% of phenotypic variance [9]. The strongest effect on English score in this study was for the same variant and in this case it explained 0.18% of observed variance (0.10% in mathematics score, **Table S4**). In this study allele score explained 0.27% of the variance in English score and 0.10% in mathematics score.

Overall, the influence of the allele score appeared to be consistent across the assessment of English and mathematics. This does have some impact on the interpretation of these effects in that this may be suggesting a basal or developmental origin rather than an acquired performance change; however it is difficult to speculate. Finer examination of these effects does suggest that to some extent the evidence for a child’s English z-score increasing with their allele score is stronger than that for mathematics. It is difficult to suggest why this might be the case, however a potential scenario may follow that if the allele score assessed here is equal in its effects across disciplines, then observed differences may be the result of measurement inconsistencies across the subjects or the result of the subjects being affected by different environmental influences. That being said, the presence of association between the same genetic variation and *both* measurements of ultimate adult educational attainment and childhood educational performance does provide another source of evidence (in addition to phenotypic correlation between these educational attainment measures) to the question of whether existing examinations are able to capture variation in early life relevant to ultimate educational attainment.

In a field where few previous GWAS have yielded evidence for common genetic contributions [21], [22] our results increase the evidence that with appropriate sample design or measurement, relationships may be found. Findings here go some way to validate the association between the three SNPs found by Rietveld et al. [9] and educational attainment suggesting that these observations are less likely to be a function of chance, artefact or residual population stratification. Furthermore, we have reinforced the assertion that it is feasible to use a more distal phenotype in order to gain a larger sample size, but have also provided the reciprocal argument in that the use of more refined measures of educational attainment can show these effects in smaller samples and that they are persistent in earlier years. It would of course be interesting to investigate whether the allele score made available from this original work can be applied to other alternative measures of educational attainment or cognitive ability in different population-based sample sets.

## Materials and Methods

### Study population and ethics statement

ALSPAC is a transgenerational longitudinal cohort study investigating factors that influence health and development. The study recruited pregnant women living in the former county of Avon, UK with estimated delivery dates between April 1991 and December 1992 [23], [24]. Children from 14,541 pregnancies were enrolled initially, rising to 15,247 pregnancies by the age of 18 years. Data has been collected for a wide range of phenotypes as well as genetic and biological samples. Information on data available can be found in the Data Dictionary (http://www.bristol.ac.uk/alspac/researchers/data-access/data-dictionary/). Ethical approval for the study was obtained from the ALSPAC Ethics and Law Committee and the Local Research Ethics Committees.

### Education data

Educational attainment was assessed by a child’s English and mathematics results in their key stage 3 national curriculum tests. Key stage 3 national curriculum tests (informally known as SATS) were statutory for children age 13 to 14 (Year 9) in maintained schools in England until 2008 [25], [26]. The children’s test results were obtained by data linkage of ALSPAC with the National Pupil Database (NPD).

The national curriculum measures achievement by levels, where the average level of attainment is level 2 at age 6 to 7, level 4 at age 10 to 11, and level 5 or 6 at age 13 to 14 [27]. The English test could only be taken at one tier whereas the mathematics test could be taken at one of four different tiers, chosen based on a child’s expected level of achievement. A child could be awarded a level N, 3, 4, 5, 6 or 7 for English and a level N, 2, 3, 4, 5, 6, 7 or 8 for mathematics. For mathematics the possible levels that they could be awarded depended on the tier that they took. A level N is given when a child does not reach the lowest level for that tier.

Due to different tiers existing for mathematics it is necessary to adjust the marks according to tier before using them in analyses. We adjusted the marks using the method described in Levăcić et al. [28]. The marks are adjusted so that, for example, a score of level 4.5 corresponds to being awarded a level 4 and having a mark that is halfway between the boundaries for a level 4 and a level 5. In creating these scores we extended the definition of the national curriculum levels to be a continuous variable taking values from 0 to 8 for English and 0 to 9 for mathematics, aligned with the discrete level values. By alignment we mean that, for example, a continuous level with a value ≥5 and <6 corresponded to the child originally being awarded a level 5. The mathematics scores were all correctly aligned with the level awarded. We dropped the English scores for 18 children whose scores were not aligned with the level that they were awarded. This may be due to errors in the data or school appeals against the level awarded resulting in a change of level. Due to a lack of normality in residuals, scores were inverse rank transformed and standardised and their z-scores were used in the analysis. We report results as standard deviation (SD) changes in SATS score throughout.

### Genetic data

9,912 ALSPAC children were genotyped using the Illumina HumanHap550 quad genome-wide SNP genotyping platform by the Wellcome Trust Sanger Institute (Cambridge, UK) and the Laboratory Corporation of America (Burlington, NC, USA). Individuals with incorrect sex assignments; extreme heterozygosity (<0.320 and >0.345 for Sanger data and <0.310 and >0.330 for LabCorp data); disproportionate levels of individual missingness (>3%); evidence of cryptic relatedness (>10% identity-by-descent) or non-European ancestry were excluded. The resulting data set consisted of 8,365 individuals. Of 609,203 SNPs, those with a minor allele frequency of <1%, a call rate of <95%, or not in Hardy–Weinberg equilibrium (p<5×10^−7^) were removed, leaving 500,527 SNPs which passed quality control. Established body mass index variants that had not been genotyped directly were imputed with MACH 1.0.16 Markov Chain Haplotyping software [13] using CEPH individuals from HapMap phase 2 as a reference set (release 22). Genotyping information for the ALSPAC mothers can be found in the Rietveld et al. supplementary material [3].

Although for the purposes of completeness we report that 500,527 SNPs passed quality control, we only analyse three SNPs rs9320913, rs11584700 and rs4851266 which allows for the follow-up of associations reported by Rietveld et al. [9].

We created an unweighted allele score from the three SNPs rs9320913, rs11584700 and rs4851266 identified in the GWAS conducted by Rietveld et al. [9]. These three genetic variants have been confirmed as single variant, genome-wide independent signals [9]. Our allele score takes the values 0, 1, …, 6. Each unit increase in the allele score corresponds to an individual having an additional educational attainment increasing allele. For the purposes of comparison, a weighted allele score was created by summing genotypic dosages at each SNP weighted via multiplying dose by beta-coefficient from a meta-analysis of Rietveld et al. [9] data (in the absence of ALSPAC study samples) and dividing this sum by the total number of alleles. There was little difference between using an unweighted or weighted allele score here since the effect sizes from Rietveld et al. [9] for each of the three SNPs are very similar. Consequently and for the purposes of interpretation, an unweighted allele score was used throughout.

### Statistical analysis

Statistical relationships between SATS z-scores and genotypic variation were undertaken using ordinary least squares (OLS) regression, taking into account covariables where necessary. Following models used in their discovery, analysis of genetic variation at the SNPs rs9320913, rs11584700 and rs4851266 both independently and within an unweighted allele score assumed an additive genetic model for each.

Since a considerable number of the children used in our analyses (3617 out of the 6208 children for English and mathematics in total) had mothers with data used in the GWAS conducted by Rietveld et al. [9] we took this into account when performing OLS regression on their children’s data. On average a child shares ∼50% of their genome with their mother so their allele score is not independent of their mother’s genotype and generating allele scores for a dataset where a related dataset was used to identify the genetic variants that make up the allele score could potentially lead to bias. Despite the fact that signals for association at the three loci employed in this study existed in the original meta-analyses in the absence of the ALSPAC mothers (**Table S1**) we further set out to take this into account by undertaking three versions of analysis.

When assessing the relationship between the allele score and SATS z-scores our exposure variable was the allele score and our outcome variables were the English and mathematics SATS z-scores. First we performed a basic regression of a child’s English z-score on that child’s allele score adjusting for sex and age at testing (in weeks). Secondly we performed regression of a child’s English z-score on that child’s allele score as previously, but in addition adjusting for their mother’s allele score composed in the same way. Thirdly we performed regression of a child’s English z-score that child’s allele score, but only using children for whom maternal genome-wide data is not available and thus whose mothers cannot have been used in the original educational attainment GWAS conducted by Rietveld et al. [9]. We repeated these three regressions with the mathematics z-score as the outcome variable, rather than the English z-score.

Whilst ALSPAC is an ethnically homogeneous population and genome-wide data are quality controlled for population structure, we also performed a further version of the first analysis adjusting for the first four PCs derived from genome-wide common variant data to assess the contribution of population structure estimated according to Price et al. [29].

We lastly assessed potential differences by sex by using a likelihood ratio test to compare a model with no interaction term to a model with an interaction term fitted between sex and allele score. We performed this test for the English and mathematics z-scores separately.

## Supporting Information

File S1 Supplementary information.
(DOCX)

Table S1 Genome-wide meta-analysis results for educational attainment in a sample excluding mothers from the Avon Longitudinal Study of Parents and Children.
(DOCX)

Table S2 Sex specific estimates of the relationship between English and mathematics SATS z-scores and allele score in the ALSPAC study.
(DOCX)

Table S3 Estimates of the relationship between English and mathematics SATS z-scores and individual SNPs rs9320913, rs11584700 and rs4851266 in the ALSPAC study.
(DOCX)

Table S4 Percentage of variation in the English and mathematics scores explained by the child’s allele score and the individual SNPs. The regression models do not include any covariables.
(DOCX)
