# Supplementary material for: Genetic Variation Associated with Differential Educational Attainment in Adults Has Anticipated Associations with School Performance in Children
Source: PLoS One. 2014 Jul 17;9(7):e100248. doi: 10.1371/journal.pone.0100248 (PMC4102483; doi:10.1371/journal.pone.0100248)
Supplement: File S1 — Supplementary information. (DOCX) [file pone.0100248.s001.docx]

**Genetic variation associated with differential educational attainment in adults has anticipated associations with school performance in children**

Mary E. Ward, George McMahon, Beate St Pourcain, David M. Evans, Cornelius A. Rietveld, Daniel J. Benjamin, Philipp D. Koellinger, David Cesarini, The Social Science Genetic Association Consortium, George Davey Smith, Nicholas J. Timpson

**Supplementary online material**

**Standard Assessment Tests (SATS)**

Within the Avon Longitudinal Study of Parents and Children, we use the Key Stage 3 (KS3) test scores from the education authorities to assess children’s school attainment. There are three assessments at KS3: English, mathematics and science. In this study we focus exclusively on English and mathematics attainment. KS3 assessments are administered at the end of Year 9, when most children are 13 or 14 years old. Children in this sample will thus have taken their KS3 tests in the period May 2005 to May 2007.

For estimation purposes, raw scores achieved in English and mathematics were recalibrated using information from the key stage level achieved and the tier at which the exam was taken (only for mathematics) to produce a score in fraction of a level (method develop by Levăcić et al. [1]). Recalibration for mathematics scores is extremely important, as it takes into account the tier at which pupils enter the exam and these tiers influence the potential maximum score for these pupils. The recalibration also means that each level equals 1.0 regardless of the level or the tier taken. Government targets suggest that pupils should make one level of progress every two years. Therefore, an estimated coefficient of 1.0 is equivalent to 24 months of ‘progress’; a coefficient of 0.5 would be equivalent to one year of progress.

For more information as to how SATS data were handled, please contact the authors.

**Collaborators**

The following people who are not listed as co-authors on this manuscript contributed to the original GWAS meta-analysis on educational attainment [[2](#_ENREF_2)], on which the present paper is based. Data access has been granted under section 4 of the Data Sharing Agreement of the SSGAC (http://ssgac.org/documents/DatasharingpolicySSGAC.pdf). The views presented in the present paper may not reflect the opinions of the individuals listed below.

Abdel Abdellaoui, Arpana Agrawal, Eva Albrecht, Behrooz Z. Alizadeh, Jüri Allik, Najaf Amin, John R. Attia, Stefania Bandinelli, John Barnard, François Bastardot, Sebastian E. Baumeister, Jonathan Beauchamp, Kelly S. Benke, David A. Bennett, Klaus Berger, Lawrence F. Bielak, Laura J. Bierut, Jeffrey A. Boatman, Dorret I. Boomsma, Patricia A. Boyle, Ute Bültmann, Harry Campbell, Christopher F. Chabris, Lynn Cherkas, Mina K. Chung, Dalton Conley, Francesco Cucca, Gail Davies, Mariza de Andrade, Philip L. De Jager, Christiaan de Leeuw, Jan-Emmanuel De Neve, Ian J. Deary, George V. Dedoussis, Panos Deloukas, Jaime Derringer, Maria Dimitriou, Gudny Eiriksdottir, Niina Eklund, Martin F. Elderson, Johan G. Eriksson, Tõnu Esko, Daniel S. Evans, Jessica D. Faul, Rudolf Fehrmann, Luigi Ferrucci, Krista Fischer, Lude Franke, Melissa E. Garcia, Christian Gieger, Håkon K. Gjessing, Patrick J.F. Groenen, Henrik Grönberg, Vilmundur Gudnason, Sara Hägg, Per Hall, Jennifer R. Harris, Juliette M. Harris, Tamara B. Harris, Nicholas D. Hastie, Caroline Hayward, Andrew C. Heath, Dena G. Hernandez, Wolgang Hoffmann, Adriaan Hofman, Albert Hofman, Rolf Holle, Elizabeth G. Holliday, Christina Holzapfel, Jouke-Jan Hottenga, William G. Iacono, Carla A. Ibrahim-Verbaas, Thomas Illig, Erik Ingelsson, Bo Jacobsson, Marjo-Riitta Järvelin, Magnus Johannesson, Peter K. Joshi, Astanand Jugessur, Marika Kaakinen, Mika Kähönen, Stavroula Kanoni, Jaakkko Kaprio, Sharon L.R. Kardia, Juha Karjalainen, Robert M. Kirkpatrick, Ivana Kolcic, Matthew Kowgier, Kati Kristiansson, Robert F. Krueger, Zóltan Kutalik, Jari Lahti, David Laibson, Antti Latvala, Lenore J. Launer, Debbie A. Lawlor, Sang H. Lee, Terho Lethimäki, Jingmei Li, Paul Lichtenstein, Peter K. Lichtner, David C. Liewald, Peng Lin, Penelope A. Lind, Yongmei Liu, Kurt Lohman, Marisa Loitfelder, Pamela A. Madden, Patrick K.E. Magnusson, Tomi E. Mäkinen, Pedro Marques Vidal, Nicolas W. Martin, Nicholas G. Martin, Marco Masala, Matt McGue, Sarah E. Medland, Osorio Meirelles, Andres Metspalu, Michelle N. Meyer, Andreas Mielck, Lili Milani, Michael B. Miller, Grant W. Montgomery, Sutapa Mukherjee, Ronny Myhre, Marja-Liisa Nuotio, Dale R. Nyholt, Christopher J. Oldmeadow, Ben A. Oostra, Lyle J. Palmer, Aarno Palotie, Brenda Penninx, Markus Perola, Katja E. Petrovic, Wouter J. Peyrot, Patricia A. Peyser, Ozren Polašek, Danielle Posthuma, Martin Preisig, Lydia Quaye, Katri Räikkönen, Olli T. Raitakari, Anu Realo, Eva Reinmaa, John P. Rice, Susan M. Ring, Samuli Ripatti, Fernando Rivadeneira, Thais S. Rizzi, Igor Rudan, Aldo Rustichini, Veikko Salomaa, Antti-Pekka Sarin, David Schlessinger, Helena Schmidt, Reinhold Schmidt, Rodney J. Scott, Konstantin Shakhbazov, Albert V. Smith, Jennifer A. Smith, Harold Snieder, John M. Starr, Jae Hoon Sul, Ida Surakka, Rauli Svento, Toshiko Tanaka, Antonio Terracciano, Alexander Teumer, A. Roy Thurik, Henning Tiemeier, André G. Uitterlinden, Matthijs J.H.M. van der Loos, Cornelia M. van Duijn, Frank J.A. van Rooij, David R. Van Wagoner, Erkki Vartiainen, Jorma Viikari, Peter M. Visscher, Veronique Vitart, Peter K. Vollenweider, Henry Völzke, Judith M. Vonk, Gérard Waeber, David R. Weir, Jürgen Wellmann, Harm-Jan Westra, H.-Erich Wichmann, Elisabeth Widen, Gonneke Willemsen, James F. Wilson, Alan F. Wright, Jian Yang, Lei Yu, Wei Zhao.

1. Levăcić R, Jenkins, A., Vignoles, A., Steele, F. and Allen, R. (2005) Estimating the Relationship Between School Resources and Pupil Attainment at Key Stage 3. DFES Research Report RR679. London: Department for Education and Skills.

2. Rietveld CA, Medland SE, Derringer J, Yang J, Esko T, et al. (2013) GWAS of 126,559 individuals identifies genetic variants associated with educational attainment. Science 340: 1467-1471.
