# Supplementary material for: Genetic Variation Associated with Differential Educational Attainment in Adults Has Anticipated Associations with School Performance in Children
Source: PLoS One. 2014 Jul 17;9(7):e100248. doi: 10.1371/journal.pone.0100248 (PMC4102483; doi:10.1371/journal.pone.0100248)
Supplement: Table S1 — Genome-wide meta-analysis results for educational attainment in a sample excluding mothers from the Avon Longitudinal Study of Parents and Children. (DOCX) [file pone.0100248.s002.docx]

**Table S1.** Genome-wide meta-analysis results for educational attainment in a sample excluding mothers from the Avon Longitudinal Study of Parents and Children.

| **Marker** | **A1** | **A2** | **Freq A1** | **Freq(SE)** | **z-score** | **pval^m^** |
| --- | --- | --- | --- | --- | --- | --- |
| rs9320913 | A | C | 0.4812 | 0.0211 | 6.735 | 1.6×10^-11^ |
| rs11584700 | A | G | 0.7804 | 0.0158 | -5.807 | 6.4×10^-9^ |
| rs4851266 | T | C | 0.3965 | 0.0178 | 5.913 | 3.4×10^-9^ |

Results are taken from an identical data set to that used by Rietveld et al. [[2](#_ENREF_2)] having removed the ALSPAC mothers’ contribution to this analysis. pval^m^ represents a p-value for the revised meta-analysis for single-point association.
