# Supplementary material for: Genetic Variation Associated with Differential Educational Attainment in Adults Has Anticipated Associations with School Performance in Children
Source: PLoS One. 2014 Jul 17;9(7):e100248. doi: 10.1371/journal.pone.0100248 (PMC4102483; doi:10.1371/journal.pone.0100248)
Supplement: Table S2 — Sex specific estimates of the relationship between English and mathematics SATS z-scores and allele score in the ALSPAC study. (DOCX) [file pone.0100248.s003.docx]

**Table S2.** Sex specific estimates of the relationship between English and mathematics SATS z-scores and allele score in the ALSPAC study.

| **Regression model** | **SNP** | **Beta** | **95%CI** | **P-value** | **Number of observations** |
| --- | --- | --- | --- | --- | --- |
| English z-score | | | | | |
| OLS of English z-score on child’s allele score (boys) | Three SNP allele score | 0.050 | 0.018, 0.082 | 0.002 | 2,989 |
| OLS of English z-score on child’s allele score (girls) |  | 0.032 | 0.004, 0.061 | 0.027 | 2,990 |
| OLS of English z-score on child’s allele score (boys) | rs9320913 | 0.079 | 0.028, 0.130 | 0.002 | 2,989 |
| OLS of English z-score on child’s allele score (girls) |  | 0.031 | -0.015, 0.078 | 0.187 | 2,990 |
| OLS of English z-score on child’s allele score (boys) | rs11584700 | 0.039 | -0.023, 0.101 | 0.215 | 2,989 |
| OLS of English z-score on child’s allele score (girls) |  | 0.031 | -0.026, 0.088 | 0.290 | 2,990 |
| OLS of English z-score on child’s allele score (boys) | rs4851266 | 0.023 | -0.029, 0.075 | 0.384 | 2,989 |
| OLS of English z-score on child’s allele score (girls) |  | 0.035 | -0.013, 0.083 | 0.158 | 2,990 |
| Mathematics z-score | | | | | |
| OLS of mathematics z-score on child’s allele score (boys) | Three SNP allele score | 0.028 | -0.004, 0.061 | 0.084 | 3,098 |
| OLS of mathematics z-score on child’s allele score (girls) |  | 0.028 | -0.001, 0.058 | 0.055 | 3,047 |
| OLS of mathematics z-score on child’s allele score (boys) | rs9320913 | 0.062 | 0.011, 0.114 | 0.018 | 3,098 |
| OLS of mathematics z-score on child’s allele score (girls) |  | 0.025 | -0.022, 0.072 | 0.298 | 3,047 |
| OLS of mathematics z-score on child’s allele score (boys) | rs11584700 | 0.020 | -0.042, 0.083 | 0.525 | 3,098 |
| OLS of mathematics z-score on child’s allele score (girls) |  | 0.037 | -0.021, 0.094 | 0.211 | 3,047 |
| OLS of mathematics z-score on child’s allele score (boys) | rs4851266 | -0.003 | -0.056, 0.049 | 0.899 | 3,098 |
| OLS of mathematics z-score on child’s allele score (girls) |  | 0.026 | -0.022, 0.075 | 0.288 | 3,047 |

Beta coefficients, 95%CIs and p-values are from a model that included age as a covariable.
