# Supplementary material for: Genetic Variation Associated with Differential Educational Attainment in Adults Has Anticipated Associations with School Performance in Children
Source: PLoS One. 2014 Jul 17;9(7):e100248. doi: 10.1371/journal.pone.0100248 (PMC4102483; doi:10.1371/journal.pone.0100248)
Supplement: Table S3 — Estimates of the relationship between English and mathematics SATS z-scores and individual SNPs rs9320913, rs11584700 and rs4851266 in the ALSPAC study. (DOCX) [file pone.0100248.s004.docx]

**Table S3.** Estimates of the relationship between English and mathematics SATS z-scores and individual SNPs rs9320913, rs11584700 and rs4851266 in the ALSPAC study.

| **Regression model** | **SNP** | **Beta** | **95%CI** | **P-value** | **Number of observations** |
| --- | --- | --- | --- | --- | --- |
| English z-score | | | | | |
| OLS of English z-score on child’s allele score (all children) | rs9320913 | 0.055 | 0.020, 0.089 | 0.002 | 5,979 |
| OLS of English z-score on child’s allele score, adjusting for maternal allele score (all children) |  | 0.067 | 0.018, 0.115 | 0.007 | 4,008 |
| OLS of English z-score on child’s allele score (children without maternal genome-wide data) |  | 0.039 | -0.020, 0.099 | 0.196 | 1,971 |
| OLS of English z-score on child’s allele score (all children), controlling for first four PCs of genetic population structure in ALSPAC |  | 0.055 | 0.020, 0.089 | 0.002 | 5,979 |
| OLS of English z-score on child’s allele score (all children) | rs11584700 | 0.035 | -0.007, 0.077 | 0.102 | 5,979 |
| OLS of English z-score on child’s allele score, adjusting for maternal allele score (all children) |  | 0.021 | -0.039, 0.080 | 0.496 | 4,008 |
| OLS of English z-score on child’s allele score (children without maternal genome-wide data) |  | 0.065 | -0.006, 0.137 | 0.073 | 1,971 |
| OLS of English z-score on child’s allele score (all children), controlling for first four PCs of genetic population structure in ALSPAC |  | 0.034 | -0.008, 0.076 | 0.109 | 5,979 |
| OLS of English z-score on child’s allele score (all children) | rs4851266 | 0.029 | -0.007, 0.064 | 0.110 | 5,979 |
| OLS of English z-score on child’s allele score, adjusting for maternal allele score (all children) |  | 0.026 | -0.023, 0.076 | 0.296 | 4,008 |
| OLS of English z-score on child’s allele score (children without maternal genome-wide data) |  | 0.013 | -0.048, 0.075 | 0.671 | 1,971 |
| OLS of English z-score on child’s allele score (all children), controlling for first four PCs of genetic population structure in ALSPAC |  | 0.029 | -0.006, 0.065 | 0.107 | 5,979 |
| Mathematics z-score | | | | | |
| OLS of mathematics z-score on child’s allele score (all children) | rs9320913 | 0.043 | 0.008, 0.078 | 0.015 | 6,145 |
| OLS of mathematics z-score on child’s allele score, adjusting for maternal allele score (all children) |  | 0.074 | 0.024, 0.123 | 0.003 | 4,106 |
| OLS of mathematics z-score on child’s allele score (children without maternal genome-wide data) |  | 0.031 | -0.029, 0.091 | 0.313 | 2,039 |
| OLS of mathematics z-score on child’s allele score (all children), controlling for first four PCs of genetic population structure in ALSPAC |  | 0.044 | 0.009, 0.078 | 0.014 | 6,145 |
| OLS of mathematics z-score on child’s allele score (all children) | rs11584700 | 0.028 | -0.014, 0.071 | 0.191 | 6,145 |
| OLS of mathematics z-score on child’s allele score, adjusting for maternal allele score (all children) |  | 0.032 | -0.028, 0.092 | 0.290 | 4,106 |
| OLS of mathematics z-score on child’s allele score (children without maternal genome-wide data) |  | 0.016 | -0.056, 0.088 | 0.660 | 2,039 |
| OLS of mathematics z-score on child’s allele score (all children), controlling for first four PCs of genetic population structure in ALSPAC |  | 0.027 | -0.015, 0.070 | 0.206 | 6,145 |
| OLS of mathematics z-score on child’s allele score (all children) | rs4851266 | 0.011 | -0.024, 0.047 | 0.534 | 6,145 |
| OLS of mathematics z-score on child’s allele score, adjusting for maternal allele score (all children) |  | 0.011 | -0.039, 0.061 | 0.675 | 4,106 |
| OLS of mathematics z-score on child’s allele score (children without maternal genome-wide data) |  | -0.003 | -0.064, 0.058 | 0.926 | 2,039 |
| OLS of mathematics z-score on child’s allele score (all children), controlling for first four PCs of genetic population structure in ALSPAC |  | 0.012 | -0.024, 0.048 | 0.520 | 6,145 |

Beta coefficients, 95%CIs and p-values are from a model that included age and sex as covariables.
