# Supplementary material for: Genetic Variation Associated with Differential Educational Attainment in Adults Has Anticipated Associations with School Performance in Children
Source: PLoS One. 2014 Jul 17;9(7):e100248. doi: 10.1371/journal.pone.0100248 (PMC4102483; doi:10.1371/journal.pone.0100248)
Supplement: Table S4 — Percentage of variation in the English and mathematics scores explained by the child’s allele score and the individual SNPs. The regression models do not include any covariables. (DOCX) [file pone.0100248.s005.docx]

**Table S4.** Percentage of variation in the English and mathematics scores explained by the child’s allele score and the individual SNPs. The regression models do not include any covariables.

| **Regression model** | **R^2^** | **P-value** |
| --- | --- | --- |
| English z-score | | |
| OLS of English z-score on allele score | 0.27% | 5.61×10^-5^ |
| OLS of English z-score on rs9320913 | 0.18% | 0.001 |
| OLS of English z-score on rs11584700 | 0.06% | 0.064 |
| OLS of English z-score on rs4851266 | 0.05% | 0.091 |
| Mathematics z-score | | |
| OLS of mathematics z-score on allele score | 0.10% | 0.012 |
| OLS of mathematics z-score on rs9320913 | 0.10% | 0.015 |
| OLS of mathematics z-score on rs11584700 | 0.02% | 0.225 |
| OLS of mathematics z-score on rs4851266 | 0.01% | 0.535 |
